# A hybrid Bi-LSTM model for data-driven maintenance planning

**DOI:** 10.1007/s43684-025-00099-9

**Published:** 2025-06-06

**Authors:** Alexandros Noussis, Ryan O’Neil, Ahmed Saif, Abdelhakim Khatab, Claver Diallo

**Affiliations:** 1 Department of Industrial Engineering, Dalhousie University, Halifax, NS Canada; 2 Laboratory of Computer Engineering (LGIPM), Lorraine University, Metz, France

**Keywords:** Deep learning, System prognostics, Selective maintenance, Reliability and maintenance optimization

## Abstract

Modern industries dependent on reliable asset operation under constrained resources employ intelligent maintenance methods to maximize efficiency. However, classical maintenance methods rely on assumed lifetime distributions and suffer from estimation errors and computational complexity. The advent of Industry 4.0 has increased the use of sensors for monitoring systems, while deep learning (DL) models have allowed for accurate system health predictions, enabling data-driven maintenance planning. Most intelligent maintenance literature has used DL models solely for remaining useful life (RUL) point predictions, and a substantial gap exists in further using predictions to inform maintenance plan optimization. The few existing studies that have attempted to bridge this gap suffer from having used simple system configurations and non-scalable models. Hence, this paper develops a hybrid DL model using Monte Carlo dropout to generate RUL predictions which are used to construct empirical system reliability functions used for the optimization of the selective maintenance problem (SMP). The proposed framework is used to plan maintenance for a mission-oriented series k-out-of-n:G system. Numerical experiments compare the framework’s performance against prior SMP methods and highlight its strengths. When minimizing cost, maintenance plans are frequently produced that result in mission survival while avoiding unnecessary repairs. The proposed method is usable in large-scale, complex scenarios and various industrial contexts. The method finds exact solutions while avoiding the need for computationally-intensive parametric reliability functions.

## Introduction

Contemporary industrial assets are becoming increasingly complex and are under constant pressure to meet the growing demands of expanding populations and the growing influence of globalization. Consequently, there is a pressing need for optimal efficiency in operations to ensure the continued success and sustainability of these assets. Maximal operation effectiveness requires these assets/systems to function reliably and consistently, thus relying upon intelligent and refined maintenance strategies. Strong maintenance policies demand striking a balance between minimizing costs, limiting maintenance times, and ensuring a high level of reliability and availability. Hence, a major topic in academic literature has been the optimization of maintenance in various contexts. Of note is the selective maintenance (SM) strategy, first proposed in [1] for mission-oriented systems encountered in many applications such as military operations, aerospace industries, and petrochemical plants. SM concerns the optimal selection of components to repair and the maintenance actions to be performed on them to guarantee maximum performance during the following mission(s) under limited maintenance resources. This resource-constrained maintenance optimization problem is known as the selective maintenance problem (SMP) [2, 3]. Often, the SMP is solved while assuming that component lifetimes are governed by probability distributions with known types and parameters. However, this assumption can prove restrictive in real-world settings where systems can be influenced by numerous environmental variables, operating conditions, and other related aspects.

In recent years, the advent of Industry 4.0 has accelerated system condition monitoring and estimation through sensors. Sensor data can be used to generate component and system health estimates/predictions through numerous methods, though the most promising technique has been the use of machine learning (ML) and deep learning (DL) methods. ML and DL methods avoid the need for assumptions about lifetime distributions, instead allowing for a direct and detailed relationship to be constructed between sensor data and component reliability estimates. For example, the prediction of the remaining useful life (RUL) for a mechanical bearing via DL was performed in [4]. Multiple literature reviews cover the application of ML and DL methods to determine component RUL and/or reliability (*e.g.*, [5, 6]). For maintenance optimization, data-driven estimates can be used as point predictions [7], probabilistic distributions [3], or even as chance constraints [8]. Most of the extant work on RUL-based maintenance optimization relies on point predictions which cannot account for prediction errors and variability.

This paper proposes a data-driven method for solving the SMP with the use of DL and Monte Carlo dropout (MCD) to develop the empirical system reliability function used for maintenance decision optimization. The paper is a significant extension of the work performed in [9], which demonstrated that a minimalist bidirectional long short-term memory model (Bi-LSTM) could be confidently used to assess bearing failures from time-series sensor data. Thus, this promising Bi-LSTM architecture is leveraged in combination with convolutional neural networks (CNNs) and dense neural networks to predict RUL values needed to solve the SMP, similar to the method proposed by [3]. The DL model is also subjected to hyperparameter tuning, which was omitted by [3]. In doing so, the proposed DL design is enhanced, and the impact of introducing CNN architecture can be quantified. Furthermore, the computationally intensive and complex reliability equations proposed in [3] are replaced with an empirical system reliability function, thus allowing the problem to be solved faster via sample average approximation (SAA). The trade-offs between loss of accuracy for using the empirical reliability function and reduced computation times are investigated via a validation experiment. The method’s capabilities and behaviors are then examined using the NASA C-MAPSS dataset [10] and an alternate prognostics dataset dealing with RUL predictions of filters [11].

The remainder of the paper is divided into six sections. Section 2 summarizes the current state of the art for ML and DL use in system prognostics, reliability modeling, the SMP and its solution methods. Section 3 describes the notation and system under consideration. Section 4 outlines the proposed integrated DL and SMP optimization framework including a description of the prediction-based empirical reliability function. Two mathematical formulations are also proposed for the SMP optimization. Multiple numerical experiments are then carried out in Sect. 5 to investigate the performance of the proposed framework. Section 6 discusses the contributions of this paper’s method and experiments. Lastly, Sect. 7 concludes the paper with a summary of its contents and a brief description of potential research extensions.

## Literature review

This literature review section covers three topics related to the issue under investigation: ML and DL methods in system prognostics, the SMP and its variants, and reliability modeling and optimization solution techniques for data-driven settings. Knowledge gaps in these themes are then highlighted, along with how this paper addresses them.

### Machine learning in system prognostics

Given the increasing prevalence of sensors for monitoring mechanical systems, the use of ML and DL methods in system prognostics has grown as a research topic. Multiple reviews have been conducted on this body of knowledge, including [5, 6]. Prognosis predictions can be separated into two main types: RUL point predictions (*i.e.,* obtaining a single RUL value) and reliability distribution predictions (*i.e.,* obtaining multiple RUL values).

Multiple methods for generating RUL point predictions have been developed, with the majority using CNNs and LSTMs. A method combining CNNs with dropout to calculate point predictions was employed in [12], attaining an accuracy higher than any prior method at the time. Similarly, in [13], two CNNs were used in series, with the first estimating the point at which a fault occurs and the second then using this estimate to inform its RUL point prediction. In contrast, an LSTM was used in [14] to predict the health state of a component, thus informing its RUL predictions. A more unorthodox method was employed in [15], wherein a two-level DL structure was developed. The lower level used LSTMs to estimate time-series data over the next few time steps based on prior data, and the upper level used LSTMs to predict RUL values based on the estimated time-series data from the lower level. The review presented in [5] noted that LSTM-based methods show strong suitability for RUL predictions and that a hybrid CNN-LSTM can automatically extract deep features, though can also be prone to overfitting. The strong RUL prediction abilities of hybrid DL methods were also highlighted in [6], though it was additionally noted that past literature tended to disregard the intensive and long computation times that can pervade DL methods.

Hybrid models blending multiple types of architecture are another common method for RUL point predictions. A hybridized DL model using an autoencoder, CNN, LSTM and deep neural network to estimate battery RULs was developed in [16]. Similarly, [17] used a hybrid CNN-LSTM model to predict RUL values, though augmented the model with Savitzky-Golay and Gaussian filters for data preprocessing and a genetic algorithm for hyperparameter tuning. In [18], a variational autoencoder for feature extraction was followed by a time-window-based sequence neural network using LSTM architecture to output RUL predictions. Meanwhile, [19] was able to generate accurate RUL point predictions based on incomplete datasets through the use of an unsupervised encoder module followed by a decoder module with a self-attention mechanism. The authors of [20] performed simultaneous predictions of the first prediction time and RUL from a temporal CNN utilizing a multi-channel attention mechanism. Lastly, [21] complemented efforts to predict RULs with a model combining particle swarm optimization with a back propagation network to estimate the time between failures for a system.

Probabilistic distribution estimation of RULs and reliability has also been investigated in some references, though less frequently than RUL point predictions. In [22], a Bi-LSTM was used for RUL predictions, and local uncertainty estimation was employed to generate probabilistic distributions for RUL intervals. An adaptive C-Transformer was used in [23] to produce component RUL point predictions, which were subsequently fed into a CNN to yield failure probabilities. The authors of [24] introduced a hybrid CNN-LSTM model to predict RUL values and then used those predictions to build probabilistic distributions of actual RUL values being less than the mission length. Monte Carlo methods were also used as a way to consider uncertainty around reliability predictions in some papers. In [25], a feedforward neural network and Bi-LSTM with MCD were combined to generate the probabilistic distributions of RULs. A hybrid DL model using Bi-LSTM and dense neural network architecture was also employed in [3] to produce RUL predictions before applying MCD to generate RUL distributions. Notably, [3] yielded DL model accuracy levels on par with, if not exceeding, those of many prior models.

### The selective maintenance problem

The SMP, first proposed in [1], considers a multi-component system that has completed its latest mission and is commencing a maintenance break of limited duration. The typical SMP decisions are which components to maintain, to what level, and by which repair channel. Following the maintenance break, the system will begin another mission, during which time it cannot be maintained and must operate at a sufficient reliability threshold [1]. Often, the SMP is optimized either to minimize cost [2] or maximize reliability [26].

The SMP has been extended in numerous directions since its inception. System configuration has been a major area of focus. The SMP was expanded in [27] to consider component ages and binary operating statuses, enabling the selection of both corrective and preventive maintenance (CM and PM) actions. Systems with multi-state components were considered in [26]. A system with multiple k-out-of-n:G subsystems in series was applied in [2]. The use of SM in the context of a fleet of systems was investigated in [3, 28]. Finally, the concept of component failure interdependence was integrated in [29].

The other key area of extension is the modeling of maintenance actions. Though often focused on minimizing cost or maximizing reliability, multi-objective optimization has been applied to the SMP in [30]. The concept of multiple imperfect maintenance (IM) levels available for each component was used in [2, 31]. Use of multiple repair channels/repairpersons was considered in [32, 33]. Stochasticity has been a major facet of research in SMP literature with stochastic mission, break, and maintenance durations researched in [34] and stochastic maintenance improvements investigated in [35]. In [36], rather than stochastic breaks, asynchronous breaks were considered for a fleet of systems with not all systems being maintained during the same break. Joint optimization has also been performed in a few papers. Maintenance plans and repairperson assignments were simultaneously optimized in [29], whereas, in [37], the maintenance plan for a fleet of systems was jointly optimized with the routes for individual repair crews. Recent and extensive reviews of the SMP can be found in [38, 39].

### Reliability modeling and solution techniques for the SMP

The most common method of reliability modeling in SMP is via parametric probabilistic lifetime distributions that usually yield time-dependent degradation, as seen in [2, 26]. Other papers, such as [40], have implemented a stochastic time-dependent health degradation process to govern component reliability. Similarly, [41] considered time-dependent multi-state systems, which were instead modeled using Markov processes.

A more modern, data-driven approach to reliability modeling was proposed in [42], using a DL model to predict component RULs and calculate the probability of mission survival according to the updated expected RULs based on the maintenance actions selected. Two major drawbacks to this method were a) a focus on series systems only, resulting in heavily simplified architecture; and, b) the use of a brute-force, full-enumeration method to solve the problem. These drawbacks limit the method’s applicability to large and complex systems. Fuzzy c-means clustering and hybrid Bi-LSTMs with dense neural networks and MCD were combined in [3] to predict component health states and RULs to inform component reliability constraints. This work was applied to a series k-out-of-n:G system, extending the work in [42] to a more complex system configuration. Data was also used to estimate reliability via Monte Carlo simulations in [43], where stochastic IM and stochastic component dependence were considered. Lastly, sensor data was used in [44] to estimate system health states and predict RUL distributions. The distributions were then sampled to generate constraints for ensuring a likelihood that a given number of components would survive until the next maintenance break, though system configuration was not considered [44].

Due to the computational difficulties in handling non-linear system reliability equations, some works have attempted to find alternative solution methods. A piecewise-linear approximation of the reliability function was developed in [34]. Multi-state SMP models have relied on heuristic optimization methods in multiple instances, including particle swarm optimization [41] and genetic algorithm [43]. The non-linear reliability of the multi-objective optimization problem provided in [30] used deep reinforcement learning to solve the SMP. In contrast, a neural network was combined with a dynamic programming algorithm to solve an SMP for large-scale problems in [45]. Finally, a hybrid heuristic method was employed in [46] wherein a discrete differential evolution algorithm searched the decision space for the ideal maintenance actions, and a deep Q-network estimated the impact of selected actions.

### Gaps in literature

Much of the past SMP work has relied upon assumed parametric lifetime distributions for reliability computation and optimization. Such assumptions greatly influence the maintenance decisions made, yet may not be representative of the actual lifetime distribution in real-world scenarios. This, in turn, may lead to significantly different maintenance plans than would be optimal. Data-driven approaches, such as the one proposed in this paper, address these issues of inaccuracy by basing their calculations on available data and making no assumptions about the system’s lifetime. Furthermore, for complex multi-component systems, the classical parametric approach yields nonlinear system reliability and optimization models that are difficult to solve. As such, classical approaches frequently require (meta)heuristics to find solutions, thus hindering their ability to achieve optimality. Given that the proposed SMP method solves to optimality, this paper’s work ensures that the best maintenance plan is found based on the data it has analyzed, thus avoiding the need for techniques such as reinforcement learning or (meta)heuristics.

Additionally, despite its demonstrable applicability to reliability modeling, the use of DL methods within the SMP scope of maintenance planning is still a relatively novel concept with several blind spots. Only three of the reviewed papers employed ML and DL methods to estimate aspects of system reliability for the SMP and contained some notable gaps. In [7], component ages were predicted before calculating reliability by assuming a probabilistic distribution form, thus risking estimation errors due to this assumption. In [42], a DL model for predicting the probability of RULs being less than a specified mission duration was used, but did not account for prediction uncertainty and only considered a basic series system, then solved the optimization via a brute-force enumeration, which cannot be scaled up. The authors of [3] did account for uncertainty in their RUL predictions and considered a far more complex system. However, they relied upon the computationally-intensive reliability function for series k-out-of-n:G systems in their optimization, thus requiring a pattern-generation method for feasibly solving their optimization. This issue was also discussed in [39], which highlighted that there is a need to develop SMP optimization methods that avoid directly calculating system reliability to enable optimization of large-scale, complex systems. It must also be noted that the use of SAA has been scant in SMP literature. Hence, this paper uses the hybrid DL model employed by [3] to optimize maintenance for a series k-out-of-n:G structure. The model is further augmented with a one-dimensional CNN, given its demonstrated strength in past related works. The model is also subjected to explicit, documented hyperparameter tuning to both further refine the design from [3] and determine whether the introduction of CNN architecture is beneficial. Using the DL model, empirical distributions are generated for the SMP model based on SAA in lieu of using the complex series k-out-of-n:G reliability function, similar to [44]. Through this method, the paper simplifies the computation of system reliability via a data-driven approach.

## Problem description

This section describes the repairable system being considered. The system of notation used is first provided. Then, a detailed description of the system and its maintenance planning framework is given along with the assumptions made.

### Notation

The notation used to define the system under consideration is as follows. Note that $E_{ij}$ is a shorthand method for referring to component *j* in subsystem *i*.

*Sets and Indices*

$\mathcal{I}$
set of subsystems, indexed by $i\in \mathcal{I}=\{1\dots I\}$
$\mathcal{J}_{i}$
set of components in subsystem *i*, indexed by $j \in \mathcal{J}_{i}=\{1\dots J_{i}\}$
$\mathcal{L}_{ij}$
set of maintenance levels available for $E_{ij}$, indexed by $l \in \mathcal{L}_{ij}=\{0\dots L_{ij}\}$
$\mathcal{N}$
set of RUL prediction samples, indexed by $n \in \mathcal{N} = \{1\dots N\}$

*Parameters*

$k_{i}$
minimum number of working components out of $J_{i}$ needed for subsystem *i* to function
$S_{ij}$
operational status of $E_{ij}$ at beginning of break
$T_{0}$
maximum break duration
$C_{0}$
maintenance budget available
$R_{0}$
minimum permitted likelihood of system surviving upcoming mission
*U*: length of upcoming mission
$c^{c}_{ijl} (c^{p}_{ijl})$
fixed cost for level *l* CM (PM) on $E_{ij}$
$t^{c}_{ijl} (t^{p}_{ijl})$
duration of level *l* CM (PM) on $E_{ij}$
$\phi _{ijl}^{n}$
survival indicator noting if $E_{ij}$ will survive upcoming mission based on RUL prediction sample *n* when maintenance level *l* is applied

*Decision Variables*

$x_{ijl}$
binary decision variable, equal to 1 when maintenance level *l* is chosen for $E_{ij}$
$y_{n}$
binary decision variable, equal to 1 when all subsystems are expected to survive upcoming mission based on sample *n* and selected maintenance plan
$R_{s}(\cdot )$
continuous decision variable representing empirical system reliability (*i.e.,* proportion of instances where system will survive upcoming mission based on RUL prediction samples and selected maintenance plan)

### System description

**Figure 1:**
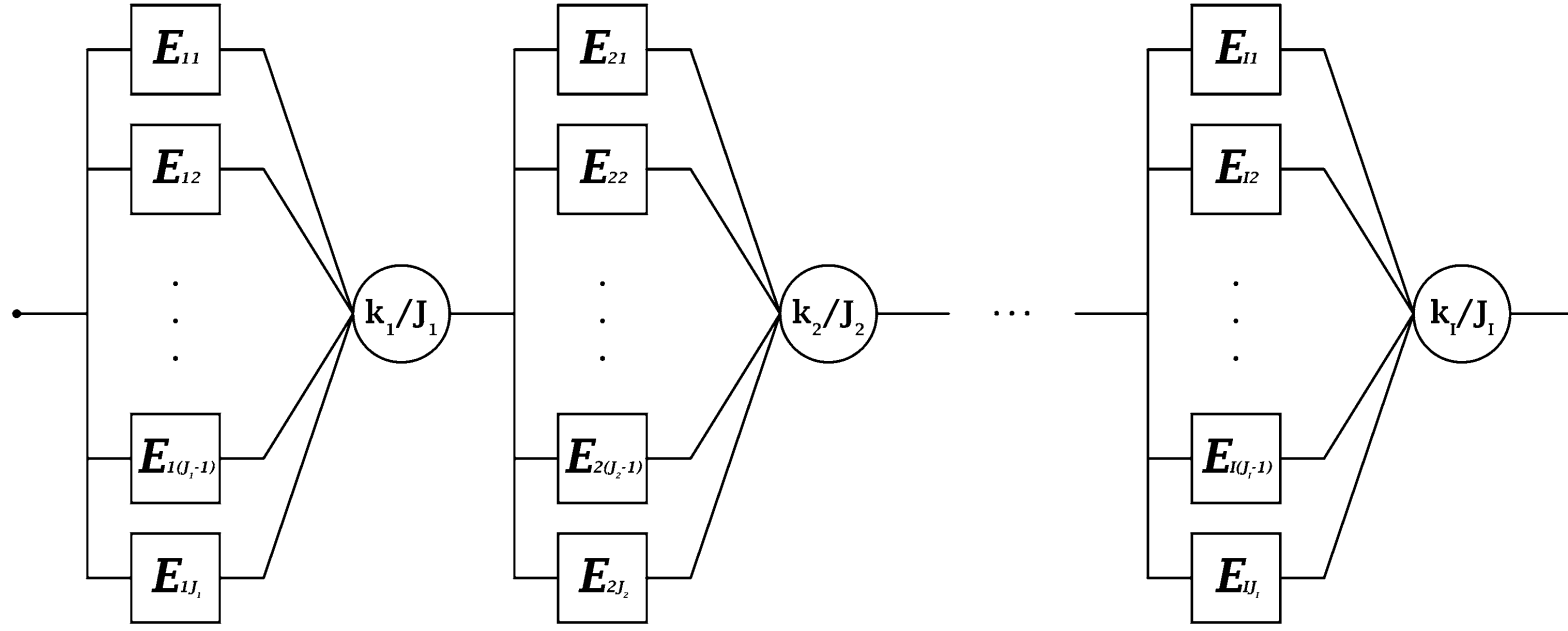
Generic RBD of the system under consideration

The system is a single mission-oriented failure-prone asset comprised of *I* subsystems in series, meaning that all subsystems must function for the system to function. Subsystem $i\in \mathcal{I}$ has a $k_{i}$-out-of-$J_{i}$:G structure, meaning that at least $k_{i}$ of its $J_{i}$ components must function for the subsystem to function. A generic version of the reliability block diagram (RBD) is presented in Fig. 1. Components (denoted as $E_{ij}$) operate statistically independently of one another and do not generally follow the same lifetime probability distribution. System and component performance are both binary: either functioning or failed.

Each component has sensors attached to it that record data on various aspects of the component’s condition, such as temperature, rotational speed, and coolant bleed [10]. One repair channel or bay is available to carry out the maintenance actions. The system has just completed a mission and is switched off to undergo maintenance during a break of length $T_{0}$ where maintenance actions may be performed with a total available maintenance budget of $C_{0}$. Maintenance cannot occur during the mission and must take place during the break. Following this break, a mission of length *U* (*e.g.*, time periods, cycles) will be performed. The status of each component $E_{ij}$ at the start of the break is described by a binary parameter as follows:

1 $$\begin{aligned} S_{ij} = \textstyle\begin{cases} 1, & \text{if $E_{ij}$ functions at the break beginning,} \\ 0, & \text{otherwise.} \end{cases}\displaystyle \end{aligned}$$

Both CM and PM actions may be performed during the break. Each maintenance action incurs a cost ($c_{ijl}^{c}$ or $c_{ijl}^{p}$) and has a duration ($t_{ijl}^{c}$ or $t_{ijl}^{p}$). Only two types of actions are considered in this problem. “Do Nothing” ($l = 0$) and “Perfect Repair/Replacement” ($l = 1$). “Do Nothing” will result in no improvement to the component’s functionality or survivability. “Perfect Repair/Replacement” will result in the component being restored to an “as good as new” state. This is reasonable and has been used by prior studies [3, 42]. Aside from ensuring that the break duration and maintenance budget are not exceeded, the system must also survive the upcoming mission with a minimum probability $R_{0}$.

## Methodology

**Figure 2:**
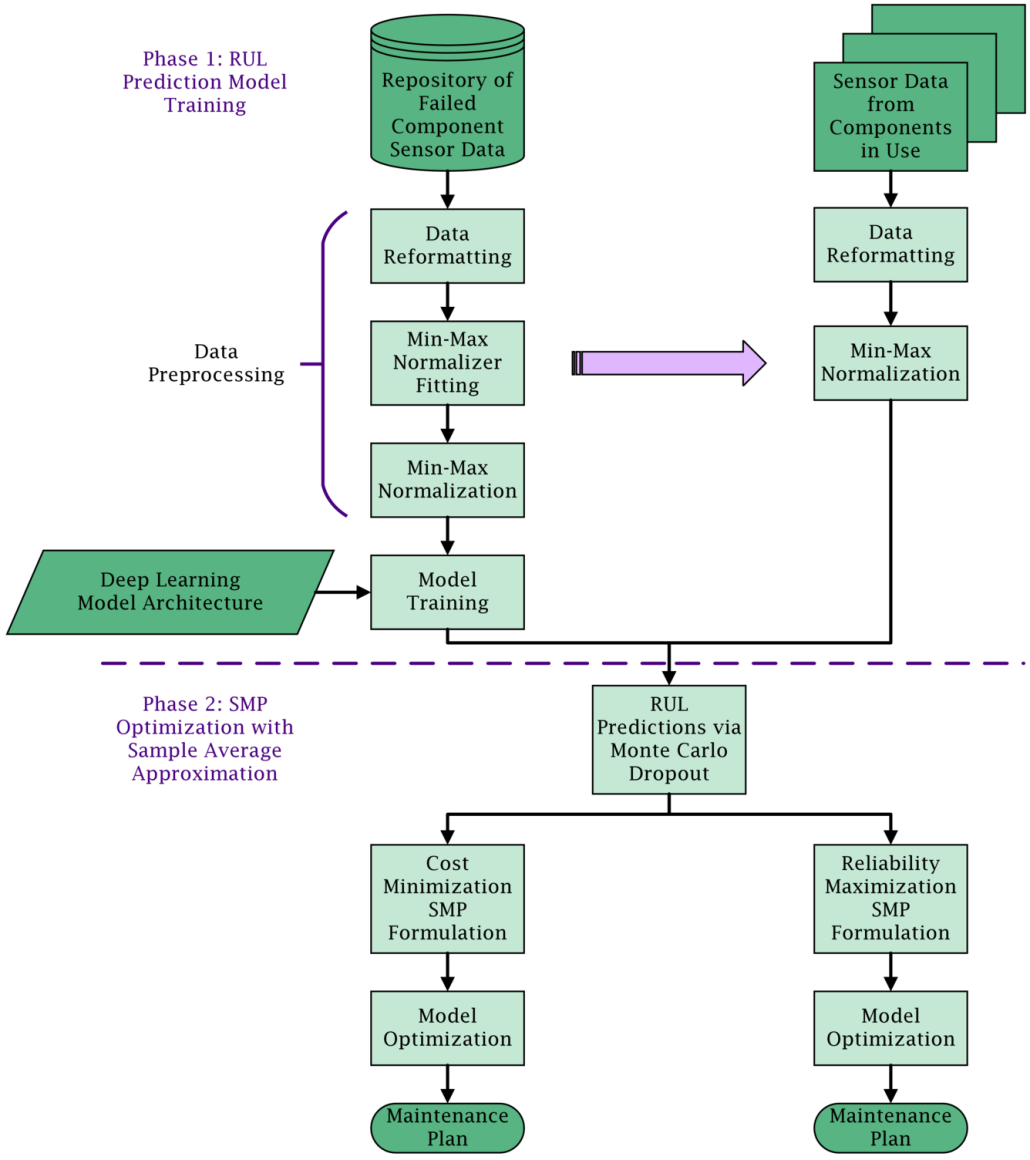
Framework of proposed two-phase method

The proposed method, described below, consists of two phases. First, a hybrid DL model is trained on past component data to predict components’ RULs based on sensor data. The trained model is used to estimate the distribution of RUL for each component via MCD. Second, the RUL prediction samples for each component are converted into a set of survival indicator parameters ($\phi _{ijl}^{n}$) indicating whether the component survives an upcoming mission according to each RUL prediction. These parameters are used to generate empirical distribution constraints for reliability modeling in an SMP formulation optimizing maintenance action selection. The overall methodological process is depicted in Fig. 2.

### RUL prediction deep learning approach

Estimating component survivability through deterministic statistical distributions is prone to errors stemming from the choice of the fitting distribution and the estimators used [3]. Thus, distribution-less methods relying directly on component health data pose a promising alternative for predicting a component’s survivability when its reliability distribution is uncertain/unknown. The collection of component health data has become increasingly easy and cheap as the use of sensors has grown in popularity. Furthermore, ML and DL methods provide the ability to better capture the relationship between various health indicators and a component’s survivability. Thus, rather than assuming the distribution parameters of each component’s reliability, this paper uses time-series sensor data collected over a component’s lifetime to estimate its RUL.

Time-series data is a common type collected in maintenance and is often analyzed via recurrent neural networks (RNNs), as they are able to capture the relationship between sequential data points, further enhancing their ability to understand the data and make predictions based on it. However, basic RNNs are known to encounter issues with understanding sequential data point inter-dependencies as the size of the sequence increases, resulting in exploding and vanishing gradients [47]. As such, LSTM models are considered a promising alternative when analyzing large sequences [48]. LSTMs can also be further broken down into their standard form of unidirectional LSTMs and bidirectional LSTMs. While standard LSTMs only analyze a data sequence in the forward direction, Bi-LSTMs analyze a sequence both in the forward and reverse directions [49]. This process, in turn, enables the model to further understand the relationship between data points, thus strengthening the model’s analytical capabilities. The strength of Bi-LSTMs was demonstrated in [9] through the use of a minimalist Bi-LSTM which yielded extremely high accuracy when handling time-series data. Thus, this paper involves the development of a hybrid DL model based on [3] utilizing Bi-LSTM and fully-connected dense neural network layers to estimate component RULs. Additionally, given the noted efficacy of CNNs for prognostics, one-dimensional CNN layers are also utilized in the DL model’s design in the hopes of further enhancing model accuracy.

This subsection presents the DL method employed by first describing data preprocessing efforts undertaken, before then detailing the DL model’s architecture.

#### Data preprocessing

Prior to analysis, data must be preprocessed to ensure it is structured appropriately for model input and able to support model training. Each data point is updated to list its RUL (*i.e.,* the number of remaining data points before the component ceases functioning). The RUL prediction is based on the method proposed in [3, 12], using a piecewise-linear target function to cap the RUL for early predictions. Preprocessing also involves the removal of data features that provide no information on component health (*i.e.,* features with constant values over time) and the scaling of data via min-max normalization to further strengthen model data analysis. Scaling involves fitting the min-max transformation to the training data before scaling testing data based on the fitted transformation [3].

Data is restructured into batches of data points. A fixed number of data points is required in each batch and is extracted from a component’s data via a sliding time window method. RNNs require data to be structured in a sequential format to process data with a temporal dimension. The data can be sampled from across the entire lifetime of recordings, or can be extracted in a batch via a sliding window. The sliding window is generally preferred since it places more value on newer information that is representative of the system’s current state, rather than past information that may not be representative of more recent deterioration in an asset’s health. The window begins at the first available data point and generates a batch. Each subsequent batch includes the next consecutive data point along with all prior data points still captured in the sliding time window. When the sliding window captures fewer data points than the fixed number needed in each batch, the missing data points are replaced by padding the front of the batch with copies of the first available data point. In its final format, data is delivered in a three-dimensional matrix of the following shape: [Number of batches across all components] × [Window size] × [Number of features].

#### Model architecture

The hybrid DL model’s architecture is as follows:

1. An input layer of the shape [Window size] × [Number of features].
2. A user-set number of one-dimensional CNN layers using rectified linear unit (ReLU) activation, each with a distinct, user-specified number of filters and kernel size.
3. A user-set number of Bi-LSTM layers, each with a distinct, user-specified number of hidden units.
4. A user-set number of fully-connected dense layers using Exponential Linear Unit (ELU) activation, each with a distinct, user-specified number of neurons.
5. A dense output layer using linear activation with a single unit.

In addition to the architecture listed above, it must be noted that dropout layers are included between each layer (except following the CNN layer) and L2 regularization is used in all layers. The architecture used for the second and third experiments of this paper is presented in Sect. 5.3, along with details on how the model is trained.

### SMP optimization with empirical reliability distribution constraints

This subsection begins by detailing the empirical distribution constraint method used for computing the system reliability from component RUL point predictions. Then, the formulations of the problem aiming to optimize the maintenance plan are presented.

#### Empirical reliability functions

Under the traditional approach based on parametric lifetime distributions, the reliability of a series system comprised of *k*-out-of-*n*:G subsystems is calculated as follows [50].

2 $$\begin{aligned} R_{s} =& \prod _{i = 1}^{I}\left [\sum _{e_{k_{i}} = 1}^{J_{i}} \sum _{e_{k_{i} - 1} = 1}^{e_{k_{i}} - 1} \dots \sum _{e_{1} = 1}^{e_{2} - 1} \left ( \prod _{v \in \{e_{1},\ldots,e_{k_{i}}\}} R_{iv}\right )\right. \\ &{}\times \left.\left (\prod _{u = 1, u \notin \{e_{1},\ldots, e_{k_{i}}\}}^{e_{k_{i}}} (1 - R_{iu}) \right )\right ] \end{aligned}$$

where $R_{ip}$ is the reliability of component $E_{ip}$. However, under data-driven approaches that use DL to predict component RULs, Equation (2) cannot be used. Thus, this paper introduces a novel approach using the predicted RUL to compute the empirical system reliability $\widehat{R}_{s}(t)$ (*i.e.,* the proportion of times the system survives out of a large number of RUL predictions).

Denote by $(X_{1},\ldots ,X_{N})$ the independent, identically distributed real random variables with the common cumulative distribution function $H(t)$. Then, by definition, the empirical distribution function $\widehat{H}_{N}(t)$ is defined as follows [51].

$$\begin{aligned} {\widehat {H}}_{N}(t) =&{ \frac {{\text{number of elements in the sample}}\leq t}{N}} \\ =&{ \frac {1}{N}}\sum _{n=1}^{N}\mathbb{I}\left ({X_{n}\leq t}\right ) \end{aligned}$$

where ${\displaystyle {\mathbb{I}(A)}}$ is the indicator of event *A*, taking the value 1 when *A* is true, and 0 otherwise. For a given *t*, the indicator ${\displaystyle \mathbb{I} \left (X_{i}\leq t\right )}$ is a Bernoulli random variable and [51] shows that ${\displaystyle {\widehat {H}}_{N}(t)}$ is an unbiased estimator for $H(t)$.

In our case, with maintenance level *l* performed, the RUL of each component $E_{ij}$ can be predicted/sampled multiple times, say *N* times, and denoted by $\text{RUL}_{ijl}^{n}$ for the $n^{\text{th}}$ prediction/sample. Hence, the empirical reliability function of component $E_{ij}$ when it undergoes maintenance level *l* is given by

3 $$\begin{aligned} \widehat{R}^{c}_{N}(U) &=\frac {1}{N} \sum _{n\in \mathcal{N}} \sum _{l \in \mathcal{L}_{ij}}x_{ijl}\cdot \mathbb{I}\left (\text{RUL}_{ijl}^{n} \geq U\right ) \end{aligned}$$

where $x_{ijl}$ is the binary decision variable equal to 1 when maintenance level *l* is carried out on component $E_{ij}$.

Let $\phi ^{n}_{ijl}$ be the random binary parameter equal to 1 if $\text{RUL}_{ijl}^{n}\geq U$, and 0 if not. Then, Equation (3) can be reformulated as

4 $$\begin{aligned} \widehat{R}^{c}_{N}(U) &=\frac {1}{N} \sum _{n\in \mathcal{N}} \sum _{l \in \mathcal{L}_{ij}}x_{ijl}\cdot \phi ^{n}_{ijl} \end{aligned}$$

For subsystem *i* to survive, at least $k_{i}$ out of $J_{i}$ components must survive the upcoming mission. The system under consideration has *I* subsystems in series, which must all survive for the system to survive. Thus, the empirical system reliability can be written as follows.

5 $$\begin{aligned} \widehat{R}_{s}(U) &=\frac {1}{N} \sum _{n\in \mathcal{N}} \mathbb{I} \left (\sum _{j \in \mathcal{J}_{i}}\sum _{l \in \mathcal{L}_{ij}}x_{ijl} \cdot \phi ^{n}_{ijl} \geq k_{i},\,\,\, \forall i\in \mathcal{I} \right ) \end{aligned}$$

#### Formulations

The SMP is commonly formulated with one of two goals: minimizing cost or maximizing reliability [39]. The cost minimization formulation, which is first presented below, aims to minimize total maintenance cost while ensuring that the maintenance break is not exceeded, only one maintenance action is performed per component (including the option to “Do Nothing”), and a minimum system reliability threshold $R_{0}$ is achieved. The system reliability constraint can be written as follows using the empirical system reliability in Equation (5).

6 $$\begin{aligned} \frac {1}{N} \sum _{n\in \mathcal{N}} \mathbb{I} \left (\sum _{j \in \mathcal{J}_{i}} \sum _{l \in \mathcal{L}_{ij}}x_{ijl}\cdot \phi ^{n}_{ijl} \geq k_{i},\,\,\, \forall i\in \mathcal{I}\right )\geq R_{0} \end{aligned}$$

Equation (6) can be equivalently rewritten without the indicator function by introducing a new binary decision variable $y_{n}$, which equals 1 if the system survives the mission based on the maintenance actions selected and the component RUL predictions $\text{RUL}_{ijl}^{n}$ in the $n^{\text{th}}$ sample.

7 $$\begin{aligned} &\sum _{j \in \mathcal{J}_{i}}\sum _{l \in \mathcal{L}_{ij}}x_{ijl} \, \phi ^{n}_{ijl} \geq k_{i} \, y_{n}, \qquad \forall n \in \mathcal{N}, i \in \mathcal{I} \end{aligned}$$

8 $$\begin{aligned} &\sum _{n=1}^{N}y_{n} \geq R_{0}\, N \end{aligned}$$

Note that $y_{n}$ can only assume the value 1 if all subsystems survive, or equivalently, if the LHS in Constraints (7) is greater than or equal to $k_{i}$ for every *i*. Otherwise, $y_{n}$ is forced to 0. Constraint (8) states that the fraction of samples in which the system survives (*i.e.,* empirical reliability) must not be less than $R_{0}$. With that, the cost-minimization formulation is given as follows.

9a $$\min_{x_{ijl},y_{n}}C=\sum_{i\in I}\sum_{j\in J_{i}}\sum_{l\in L_{ij}}\left(c_{ijl}^{c}\left(1-S_{ij}\right)+c_{ijl}^{p}S_{ij}\right)x_{ijl}$$

9b $$\begin{array}{rlll} \text{s.t.:}\; & & & \\ & \sum_{j\in J_{i}}\sum_{l\in L_{ij}}x_{ijl}\;\phi_{ijl}^{n}\geq k_{i}\;y_{n},\;\forall n\in N,i\in I \end{array}$$

9c $$\begin{aligned} & \sum _{n \in \mathcal{N}} y_{n} \geq R_{0} \, N \end{aligned}$$

9d $$\sum_{i\in I}\sum_{j\in J_{i}}\sum_{l\in L_{ij}}\left(t_{ijl}^{c}\left(1-S_{ij}\right)+t_{ijl}^{p}\;S_{ij}\right)x_{ijl}\leq T_{0}$$

9e $$\begin{aligned} & \sum _{l \in \mathcal{L}_{ij}}x_{ijl} = 1,\qquad\qquad \qquad \forall i \in \mathcal{I}, j \in \mathcal{J}_{i} \end{aligned}$$

9f $$\begin{aligned} & x_{ijl}, y_{n} \in \left \{0, 1\right \},\qquad \qquad \quad \forall i , j , l , n \end{aligned}$$

The objective function given in Equation (9a) minimizes the total maintenance cost, which is the sum of all CM and PM costs incurred. The empirical distribution constraints used to model reliability based on RUL predictions are presented in Constraints (9b) and (9c). Constraint (9d) ensures that the total maintenance time does not exceed the allotted break duration. Constraints (9e) ensure that only one maintenance action is selected for each system component (including the “Do Nothing” option). Finally, Constraints (9f) define the binary domain of the decision variables. This is a binary integer program that can be handled by off-the-shelf optimization solvers.

Similarly, the formulation for maximizing the system reliability given a total maintenance budget $C_{0}$ is presented below.

10a $$\max_{x_{ijl},y_{n},R_{s}}R_{s}$$

10b $$\begin{array}{rlll} \text{s.t.:} & & & \\ & \sum_{j\in J_{i}}\sum_{l\in L_{ij}}x_{ijl}\;\phi_{ijl}^{n}\geq k_{i}\;y_{n},\;\forall n\in N,i\in I \end{array}$$

10c $$\begin{aligned} & \sum _{n \in \mathcal{N}} y_{n} \geq R_{s} \, N \end{aligned}$$

10d $$\sum_{i\in I}\sum_{j\in J_{i}}\sum_{l\in L_{ij}}(c_{ijl}^{c}\;(1-S_{ij})+c_{ijl}^{p}\;S_{ij})\;x_{ijl}\leq C_{0}$$

10e $$\sum_{i\in I}\sum_{j\in J_{i}}\sum_{l\in L_{ij}}(t_{ijl}^{c}\;(1-S_{ij})+t_{ijl}^{p}\;S_{ij})\;x_{ijl}\leq T_{0}$$

10f $$\begin{aligned} & \sum _{l \in \mathcal{L}_{ij}}x_{ijl} = 1,\qquad\qquad \qquad \forall i \in \mathcal{I}, j \in \mathcal{J}_{i} \end{aligned}$$

10g $$\begin{aligned} & x_{ijl}, y_{n} \in \left \{0, 1\right \},\qquad \qquad \quad \forall i, j, l, n \end{aligned}$$

10h $$\begin{aligned} & 0 \le R_{s} \le 1 \end{aligned}$$

The objective function in (10a) maximizes the empirical system reliability denoted here by the decision variable $R_{s}$ and computed by constraints (10b) and (10c) as the proportion of RUL prediction samples where the system survives the upcoming mission based on the selected maintenance plan. Constraint (10d) ensures that the maintenance budget $C_{0}$ is not exceeded. Constraints (10e), (10f), and (10g) serve the same respective purposes as Constraints (9d), (9e), and (9f) in the cost minimization formulation. Lastly, Constraint (10h) defines the domain of the $R_{s}$ decision variable, though it is redundant in this formulation.

## Numerical experiments and results

This section details the experiments performed to assess the proposed method’s benefits and drawbacks. A validation experiment is first conducted to compare the data-driven SMP formulations to the traditional methods using assumed parametric lifetime functions. This validation is then followed by two experiments to demonstrate the capabilities of the proposed method’s DL model. The NASA C-MAPSS dataset is used in these experiments and is described along with scenario design and DL model hyperparameters before detailing the experiments. Additionally, to illustrate the method’s applicability to various industrial settings, an alternate dataset for filter RUL prediction is used in the aforementioned maintenance scenario.

### Experiment 1: validation of the new SMP formulation

To understand the accuracy of the empirical reliability distribution, the SMP formulation is tested with the scenario and parameters used in [2] for their fifth experiment. This experiment considers a series k-out-of-n:G system with an upcoming mission length $U=8$, a break duration $T_{0}=100$, and a budget $C_{0}=180$. Rather than using sensor data, [2] assumed that each component’s lifetime followed a Weibull distribution, though each component’s distribution parameters could differ. “Do Nothing”, “Perfect Repair”, “Minimal Repair”, and “Imperfect Repair” actions were permitted in [2]. To account for the effect of each action, the age of repaired components was adjusted via age reduction coefficients. Optimizing their SMP model yielded a system reliability $R_{s} = 0.8138$ with a computation time of 1.92 seconds.

**Figure 3:**
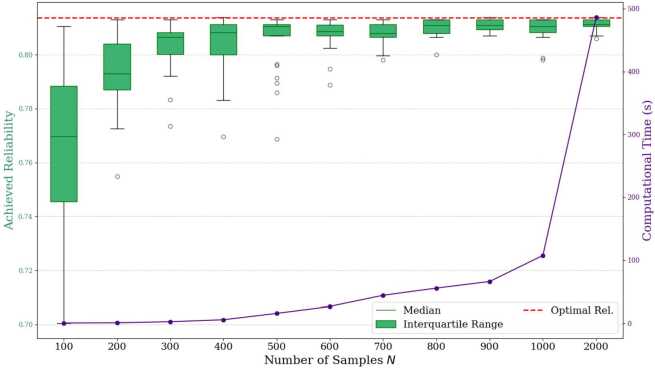
Validation experiment performance metrics when varying sample size *N*

The lifetime distributions and parameters described above are used for this set of experiments. RUL predictions are sampled directly from the assumed component lifetime distributions based on their current ages. The number of samples taken is varied between 100 and 2000 and the formulation is optimized 25 times for every sample size with new samples taken every time. Figure 3 and Table 1 present the achieved reliability (average and standard deviation) and computation time for each sample size. The gap listed in Table 1 is the percent difference between the average system reliability achieved ($\widehat{R}_{s}$) and the parametric reliability ($R_{s}$) achieved via the classical method (*i.e.,* $\text{Gap}= \frac{R_{s}-\widehat{R}_{s}}{R_{s}}$).

**Table 1:** Validation experiment performance results when varying sample size *N*

Sample	Achieved Reliability				
Size *N*	Average	Std. Dev.	Gap (%)	$\bar{\gamma}_{25}$ (%)	CPUt (s)
100	0.768	0.028	5.58	63.36	0.6
200	0.793	0.014	2.56	67.93	1.0
300	0.803	0.009	1.35	71.94	2.7
400	0.803	0.012	1.29	72.83	5.8
500	0.805	0.011	1.07	72.91	15.9
600	0.808	0.006	0.74	73.94	26.6
700	0.808	0.004	0.70	72.57	44.1
800	0.810	0.003	0.45	73.43	55.5
900	0.811	0.002	0.35	73.83	66.0
1000	0.810	0.004	0.50	73.20	107.5
2000	0.811	0.002	0.29	76.00	486.2

A stability metric (*γ*) is also included in Table 1 to assess the consistency in the maintenance plans generated (*i.e.,* the number of maintenance actions that tend to remain the same with each random set of RUL samples). Each optimal maintenance plan is structured as a vector of binary values representing the maintenance levels chosen for each component (*i.e.,* 1 if the corresponding maintenance level is selected for the component, 0 otherwise). When comparing two different maintenance plans, the $l_{1}$-norm, defined as the sum of the absolute value of differences between the two plans, can be used as a measure of their dissimilarity. Let two different maintenance plans, denoted $Plan^{a}$ and $Plan^{b}$, be represented as two vectors of binary decision variables $x_{ijl}^{a}$ and $x_{ijl}^{b}$, respectively. Thus, $l_{1}$-norm $= \sum _{i,j,l}\left |x_{ijl}^{a} - x_{ijl}^{b} \right |$. Then,

$$ \gamma _{a,b}= 1 - \dfrac{\sum _{i,j,l}\left |x_{ijl}^{a} - x_{ijl}^{b} \right |}{2\cdot \sum _{i} J_{i}} $$

where the number of differences is divided by the potential total number of differences between the two vectors. Note that the SMP formulations proposed in this paper include a constraint requiring exactly one maintenance action to be selected for each component in the system. Thus, each change in component maintenance level yields a difference of 2 (*i.e.,* one binary variable goes from 0 to 1, and another goes from 1 to 0). $\sum _{i} J_{i}$ is the total number of components in the system. In lay terms, $\gamma _{a,b}$ is the proportion of system components that have the same maintenance action selected between plans *a* and *b*. This metric can be calculated for every pair of the *m* maintenance plans obtained from the *m* trial runs. The mean number of maintenance levels that remain the same between plans is calculated as $\bar{\gamma}_{m}$.

$$ \bar{\gamma}_{m} = \dfrac{\sum _{a} \sum _{b} \gamma _{a,b}}{\frac{m\cdot (m-1)}{2}} $$

This averaged value is a measure of solution stability, with a higher value being more desirable.

**Figure 4:**
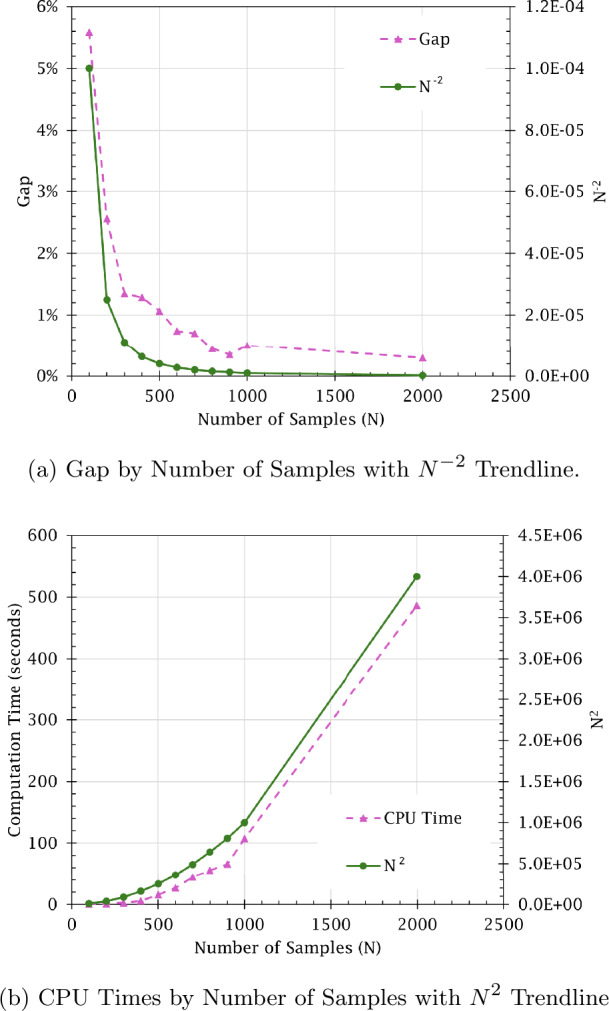
Gap and CPU times by number of samples (*N*) with trendlines

**Figure 5:**
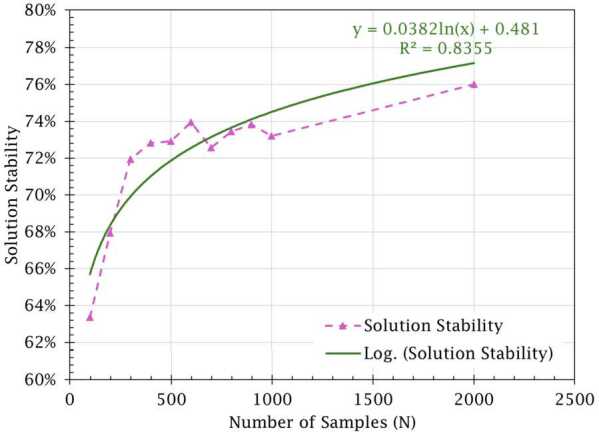
Solution stability by number of samples with logarithmic trendline

The results show that the proposed sampling-based approach is a good approximation to the classical parametric approach. With a number of samples of 600 or greater, the gap to optimality is less than 1%. The results indicate that the proposed SAA method is able to achieve near-optimal results. As the number of samples increases, the model approaches optimality with less deviation in solution results. Furthermore, solution stability also improves as the number of samples increases, increasing by about 13 percentage points when comparing sample sizes of 100 and 2000. This highlights that, as more samples are taken, the number of components that would have their selected maintenance action changed between plans decreases since the uncertainty in the component RULs also decreases. However, the average runtime increases drastically with the number of samples taken. These relationships are visualized in Figs. 4a and 4b. As the graphs illustrate, the relationship between optimality gap and CPU time is inversely proportional. Thus, there is a trade-off between taking RUL prediction samples to approach optimality with more certainty and producing results in a feasible time. Furthermore, Fig. 5 illustrates an inverse logarithmic relationship between stability and number of samples taken. This relationship indicates that, as with the optimality gap, there are diminishing returns for continuing to increase the sample size.

The proposed method achieved near-optimal results with minimal uncertainty in 24.9 seconds on average when taking 600 RUL samples, indicating that the model does not require an extremely large number of samples to yield good results. A sample size of 600 RUL samples is also where the second-best stability is achieved, although this is likely due to stochasticity since the stability is quite similar for the samples sizes between 400 and 1000. Inherent to the use of an approximation method, it is impossible to guarantee the selection of an optimal maintenance plan unless an infinite number of samples are taken. However, it is observed that the error (standard deviation) in maintenance plan efficacy due to sampling quickly shrinks in this case once at least 600 samples are taken. Past this point, the method is able to consistently yield maintenance plans with near-identical effects on reliability in comparison to the benchmark method from [2]. Thus, it has been determined that the proposed method’s use of empirical reliability functions can accurately model reliability distributions. Furthermore, these results are produced without assuming the lifetime distribution of components, which, as stated previously, is useful when having to base analyses on a limited dataset.

### The NASA C-MAPSS dataset

The NASA C-MAPSS dataset [10], a popular choice for mechanical system diagnostics and prognostics research due to its quality and volume of data, is used for the second and third experiments in this paper. The dataset contains time-series sensor data sequences for commercial turbofan engines generated by the C-MAPSS simulation tool. The features provided are three operational settings and 21 sensors. A damage propagation model simulates the engine’s failure until it reaches a random deterioration state and degradation parameters. The final data is modified to include noise from real-world sensor data. The dataset is divided into training and testing subsets. The training subset provides engine data until failure, while the testing subset provides pruned simulation data before engine failure.

For this paper’s experiments, the turbofan engines are treated as a set of generic system components. Each engine’s time-series is considered a component’s lifetime data with a subset of 100 turbofan engine simulations (train_FD001) from the training data subset being selected for the experiments. The reasoning behind using training data is that the experiments can be more carefully tuned for certain scenarios, as each engine’s simulation contains all data until failure, allowing data to be pruned as desired. Additionally, as mentioned in Sect. 4.1.1, unnecessary and irrelevant data features are culled, resulting in all operational settings being omitted, along with sensors 1, 5, 6, 10, 16, 18, and 19.

### Data batching and hyperparameter tuning

To test the efficacy and performance of the proposed method, test scenarios must be established. A moderately large system size of 20 components is selected with the structure depicted in Fig. 1 based on the subsystem architecture documented in Table 2.

**Table 2:** Subsystem Architecture for Experiments 2 and 3

Subsystem (*i*)	Number of Components in Subsystem ($J_{i}$)	Number of Components Needed to Survive ($k_{i}$)
1	4	2
2	7	3
3	9	4

Each component is assigned a different turbofan engine’s dataset, an age (in cycles), and a health status ($S_{ij}$) when the maintenance break is entered. The 100 engines selected for use in these experiments are randomly shuffled and split into five batches of 20 engines each. This allows for five different batches of data to be available for test scenarios. The model is trained and validated on four out of the five engine data batches and then has 20 engines to use as test data. These 20 engines used as test data represent the data for the components in the system described in the previous paragraph.

For each component, the respective engine simulation data is trimmed at the component’s age. Window length (*WL*) is used as the time-series data sequence length. Thus, from the remaining data, the last *WL* data points are used as the respective component’s time-series data sequence for predicting its RUL. If fewer than *WL* data points are available, all available points are taken and the first data point is used to pad the front of the data sequence. These data batches are used by the proposed hybrid DL model with MCD (CNN-Bi-LSTM-Dense-MCD) to generate RUL prediction samples. The samples are then converted into the survival indicator values ($\phi _{ijl}^{n}$) used in the maintenance optimization models.

The Adam optimizer is used to train the model with a user-set learning rate and the loss function selected for training is the mean squared error (MSE). An adaptive learning rate mechanism is implemented for training based on the lack of improvement in the MSE for validation data (a subset of training data). If a minimum MSE reduction is not achieved with the validation data over a set number of epochs, the learning rate is reduced by a specified factor with a minimum permissible learning rate for continuing training.

The study in [3] did not document any explicit hyperparameter tuning, instead it used hyperparameters from prior literature. This paper’s work involves hyperparameter tuning to further enhance the accuracy and effectiveness of the proposed DL model and determine whether augmenting with CNN architecture is worthwhile. Nine hyperparameters are selected for tuning in the following order:

1. Learning rate
2. Batch size (default = 128)
3. Window length (default = 50)
4. Minimum required improvement for the learning rate reduction mechanism (default = 10)
5. Number of filters in the CNN layer (default = 32)
6. CNN layer kernel size (default = 5)
7. Number of hidden dimensions in each Bi-LSTM layer (default = 20 and 20)
8. Number of units in the fully-connected dense layer (default = 100)
9. Dropout rate (default = 0.5)

For each hyperparameter setting, two models are trained, each with a different batch of training and test engines. Each model is trained five separate times to gather average performance metrics and mitigate the impact of randomness in model training. Mean absolute error (MAE), mean squared error (MSE), root mean squared error (RMSE) and model training and testing times are all recorded.

Additionally, three metrics are often used to assess the performance of ML models using the C-MAPSS dataset based on the difference between the average predicted RUL for a test sequence and the actual RUL: an alternate version of root mean squared error (RMSE_C_), a scoring function (SC) and accuracy (AC) [3, 25, 49]. These metrics are calculated based on a set of data samples $\mathcal{S}$ with $\widehat{\text{RUL}}_{s}^{n}$ representing the *n*th prediction of a component’s RUL based on data sample *s*, $\text{RUL}_{s}$ representing the actual component RUL, and $E_{s}$ representing the error between the average RUL prediction for data sample *s* and its actual RUL. The equations used are as follows:

11 $$\begin{aligned} &E_{s} = \left ( \frac{1}{|\mathcal{N}|}\sum _{n \in \mathcal{N}} \widehat{\text{RUL}}_{s}^{n} \right ) - \text{RUL}_{s} \end{aligned}$$

12 $$\begin{aligned} & \text{RMSE}_{\text{C}} = \sqrt{ \frac{\sum _{s \in \mathcal{S}} (E_{s})^{2}}{|\mathcal{S}|}} \end{aligned}$$

13 $$\begin{aligned} & \text{sc}_{s} = \textstyle\begin{cases} e^{\frac{-E_{s}}{13}}\, \text{if} \, E_{s} \leq 0, \\ e^{\frac{E_{s}}{10}}\, \text{if} \, E_{s} \geq 0 \end{cases}\displaystyle \end{aligned}$$

14 $$\begin{aligned} & \text{SC} = \sum _{s \in \mathcal{S}}\text{sc}_{s} \end{aligned}$$

15 $$\begin{aligned} & \text{AC} = \frac{1}{|\mathcal{S}|}\sum _{s \in \mathcal{S}} \mathbb{I}\left (-13 \leq E_{s} \leq 10 \right ) \end{aligned}$$

SC is skewed based on the number of samples tested. Thus, only AC is provided to enable an additional metric for comparison across the hyperparameter settings. All results from hyperparameter tuning are presented in Table 3 with the settings chosen for the model highlighted in gray. The hyperparameter settings chosen are based on two key factors. First, if the majority of metrics perform best with the setting, this is often an indication that the setting is ideal. Second, if the model runtime increases drastically with the setting, an alternate setting may be selected if performance is not significantly impacted. This is the case for the batch size hyperparameter, as a batch size of 64, while performing best, would require significantly more time to perform future hyperparameter tuning. As such, a batch size of 128 is viewed as an acceptable trade-off to avoid major runtime increases.

**Table 3:**
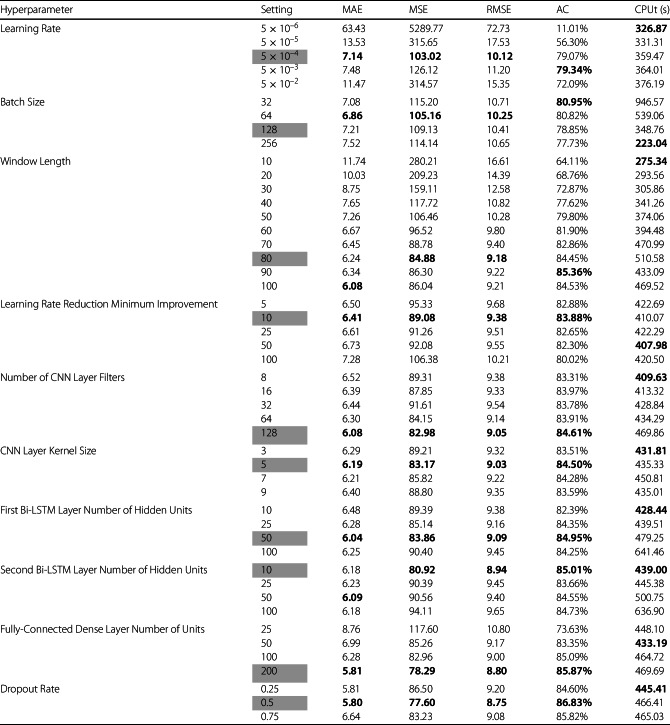
Hyperparameter tuning results (best values in bold, chosen settings in gray)

**Figure 6:**
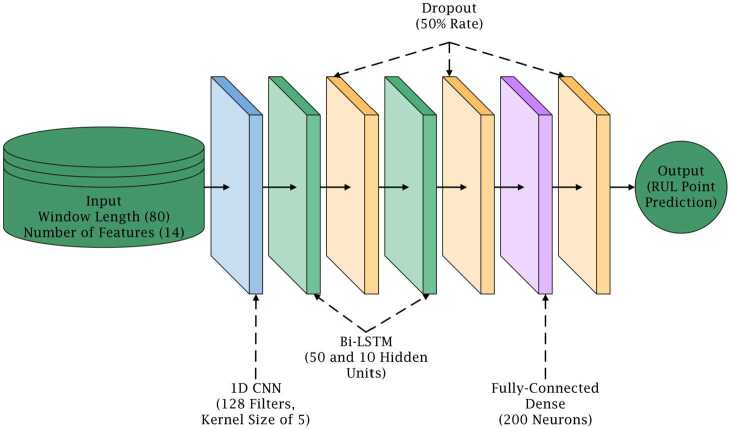
Deep learning model architecture for experiments 2, 3, and 4

The finalized list of hyperparameters used for model training are provided below and the final model’s architecture is provided in Fig. 6.

- Window length (*WL*) = 80 time steps
- Maximum RUL value = 125 cycles
- Number of epochs = 100
- Batch size = 128
- Training/Validation split = 75/25
- Number of CNN layers = 1
- Number of CNN layer filters = 128
- CNN layer kernel size = 5
- Number of Bi-LSTM layers = 2
- Number of hidden units in Bi-LSTM layers = [50, 10]
- Number of fully connected dense layers = 1
- Number of dense layer units = 200
- Dropout rate = 0.5
- Initial learning rate = 0.0005
- L2 regularization strength (*μ*) = 0.001
- Minimum MSE improvement required for adaptive learning rate = 10
- Adaptive learning rate patience = 10 epochs
- Adaptive learning rate reduction coefficient = 0.5
- Minimum permissible learning rate = $1\times 10^{-10}$

The number of RUL predictions generated through MCD (*N*) is also varied for the finalized model architecture to understand its impact on MAE, MSE, RMSE, AC, and model runtime. Five models are trained and tested, each using a different test engine batch. Each model is tested with each sample size and the results of these tests are presented in Table 4.

**Table 4:** Finalized model test data results with varying MCD sample size (best values in bold)

MCD Sample Size	MAE	MSE	RMSE	AC	CPUt (s)
100	6.13	81.82	9.01	**85.69%**	**19.6**
250	6.11	81.19	8.97	85.56%	48.1
500	6.10	80.92	8.96	85.63%	91.0
1000	6.10	80.93	8.96	85.62%	189.1
2000	**6.09**	**80.84**	**8.95**	85.62%	359.6

Metrics improve by very little as the sample size increases, indicating that the baseline model is strong and does not require a large number of RUL predictions to produce close estimates. Interestingly, the accuracy metric achieves its best result with the lowest sample size. This may be due to stochasticity in model training and/or the MCD predictions. The sample size of 1000 is selected as the baseline setting to ensure that model estimates are near their best performance without incurring major runtime increases.

For maintenance optimization, the upcoming mission length is set to $U=80$ cycles, the total break duration is set to $T_{0}=50$ time units, and the total budget available is set to $C_{0}=150$. The minimum reliability threshold is set to $R_{0}=0.90$ for the cost minimization model. The number of RUL prediction samples is set to $N=1000$ for each component. This scenario considers fewer maintenance levels than [2] since the traditional age reduction coefficient method is not applicable. The effects of imperfect maintenance on a component cannot be quantified since that data has not been provided in the NASA C-MAPSS dataset. The application of imperfect maintenance in a data-driven method requires experimental results from attempting such repairs in a test environment. As such, only the “Do Nothing” and “Perfect Repair/Replacement” actions are considered, and thus, a greater number of samples can be taken while retaining feasible computation times. The maintenance action cost and time parameters used are provided in Table 5. All experiments are executed in Google Colab on a Tesla T4 GPU.

**Table 5:** Subsystem maintenance costs and times for experiments 2 and 3

Subsystem (*i*)	$c^{c}_{ij1}$	$c^{p}_{ij1}$	$t^{c}_{ijl1}$	$t^{p}_{ij1}$
1	14	10	8	4
2	20	12	5	2
3	10	7	4	3

### Experiment 2: performance of the RUL prediction model

Five models are trained for this experiment, each being trained on 80 of the 100 engines provided in the NASA C-MAPSS dataset and tested with the remaining set of 20 engines. For each of the five models, Table 6 shows the following performance metrics: MAE, MSE, and RMSE based only on the 20 test engines from the train_FD001 dataset, as well as RMSE_C_, SC, and AC based on the test_FD001 dataset, and model training time. To compare this model’s performance against prior works, Table 7 lists RMSE_C_, SC, and AC for various past models, with this paper’s best model metrics provided in the last row of the table. The proposed DL model is found to have an acceptable performance level, yielding an RMSE_C_ and SC that are among the best, and achieving the best AC. These results show that the introduction of the CNN architecture and the hyperparameter tuning have enhanced the performance of the model proposed by [3].

**Table 6:** Finalized deep learning model performance metrics

Model	MAE	MSE	RMSE	RMSE_C_	SC	AC	Model Training Time (s)
1	5.45	74.64	8.64	12.81	299.36	72%	296.1
2	7.16	101.08	10.05	11.80	194.79	75%	426.5
3	6.22	99.75	9.99	11.49	203.77	75%	437.1
4	5.77	76.11	8.72	12.53	242.96	73%	424.4
5	5.08	63.07	7.94	11.91	218.32	72%	436.6
*Average*	5.94	82.93	9.07	12.11	231.84	73%	404.2

**Table 7:** State-of-the-art prognostic DL model comparison (best values bolded)

Model	RMSE_C_	SC	AC
SBI [52]	13.58	228.00	67%
1-FCLCNN-LSTM [53]	**11.17**	204.00	–
PGRU [54]	12.39	–	–
BDL [25]	12.70	234.90	70%
Multi-channel CNN w/ MCD [55]	11.81	–	–
ATCN [56]	**11.48**	**194.25**	–
BGT [57]	12.09	262.67	–
ISG-McMsDCNN-LSTM [17]	**10.43**	**162.63**	–
Bi-LSTM-MCD [3]	**11.64**	214.85	**74%**
CNN-Bi-LSTM [58]	13.22	232.24	–
CNN-Bi-LSTM-Dense-MCD (Model 2, this paper)	**11.80**	**194.79**	**75%**

**Figure 7:**
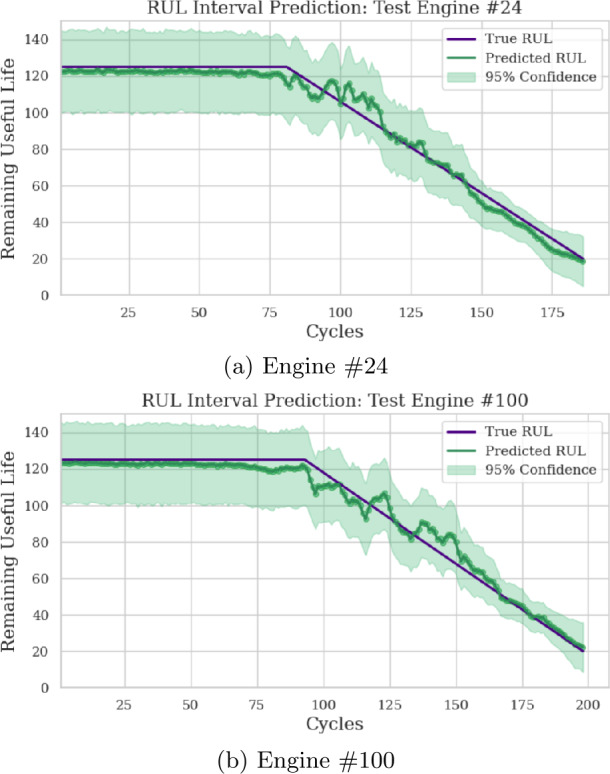
RUL point prediction accuracy visualization for two test engines

**Figure 8:**
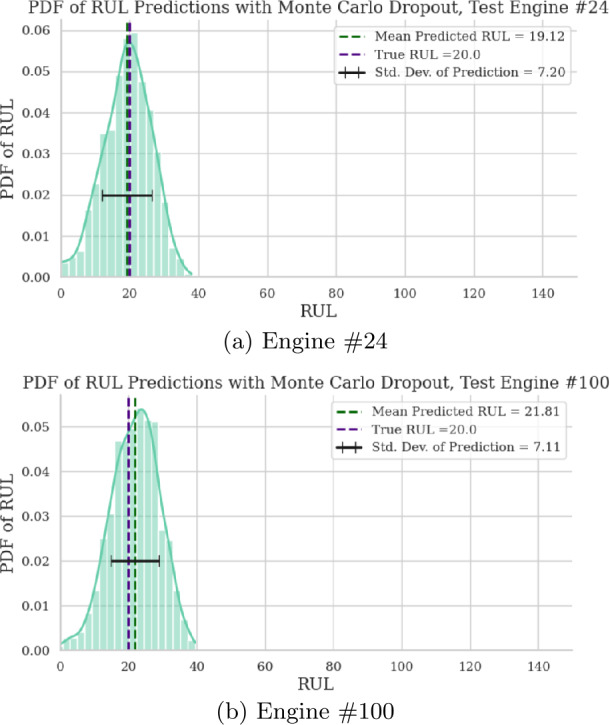
RUL prediction distribution for two test engines

Furthermore, the RUL point predictions for engines 24 and 100 from the test_FD001 dataset are depicted in Fig. 7. The plots show the true RUL, mean predicted RUL, and the 95% confidence interval around the mean prediction. The mean predicted RUL closely matches the true RUL, especially as the true RUL tends to 0. Moreover, the 95% confidence interval consistently includes the true RUL. Lastly, Fig. 8 shows the distribution of RUL predictions at the final point for each of the two test engines.

After the CNN-Bi-LSTM-Dense-MCD is shown to provide accurate RUL estimates, its predictions are used to optimize the selective maintenance plans under multiple trials using the experimental parameters outlined above. For each batch of engines, 10 trials are run where each engine’s age and health status are randomized. Ages are randomized between one cycle and the respective engine’s failure age. In total, 50 trials (5 batches × 10 trials per batch) are tested to find the optimal maintenance plan based on both objective functions: minimizing cost and maximizing reliability.

Table 8 varies the number of RUL predictions taken for each component and reports the average maintenance plan cost, duration, and empirical reliability based on the plans selected in the optimizations, along with the proportion of trials survived based on the selected plans. The average trial time required to gather all RUL prediction samples and optimize both maintenance plans is also provided. It is observed that increasing sample sizes results in performance convergence. Once a sample size of 500 is reached, the cost minimization plan’s cost no longer improves and the proportion of trials survived in the reliability maximization plan reaches 1.00. As such, there is no need to take an extremely large number of samples to yield strong SMP plans. For the remainder of the experiments, a sample size of 1000 is used to ensure strong results with minimal runtime impacts.

**Table 8:** Optimal maintenance plan metrics: Case of 50 trials with varying MCD sample size

MCD Sample Size	Objective Function	Average Cost	Average Duration	Average Empirical Reliability	Fraction of Trials Survived	Average Trial CPUt(s)
100	Cost Min.	41.70	13.52	0.9532	0.86	8.3
	Rel. Max.	87.04	28.14	1.0000	0.98	
250	Cost Min.	41.56	13.46	0.9543	0.86	18.7
	Rel. Max.	85.08	27.62	1.0000	0.96	
500	Cost Min.	41.42	13.40	0.9668	0.86	37.1
	Rel. Max.	89.18	29.44	1.0000	1.00	
1000	Cost Min.	41.42	13.40	0.9574	0.86	73.1
	Rel. Max.	89.14	29.32	1.0000	1.00	
2000	Cost Min.	41.42	13.40	0.9567	0.86	145.3
	Rel. Max.	85.28	28.64	1.0000	1.00	

Finally, Tables 9 and 10 present the average classification metrics for the reliability maximization and cost minimization optimization plans, respectively. The classification metrics are based on maintenance decisions at the subsystem level. True positives (TP) represent subsystems that are not going to survive the upcoming mission and receive adequate maintenance, resulting in mission survival. False positives (FP) represent subsystems that are going to survive, yet still receive maintenance. True negatives (TN) represent subsystems that are going to survive and do not undergo any maintenance. False negatives (FN) represent subsystems that are not going to survive and do not receive enough maintenance to result in mission survival. These confusion matrix results can be summarized in a number of key metrics. Precision $\left (\tfrac{TP}{TP + FP}\right )$ represents the model’s tendency to perform only critical maintenance on subsystems that are not going to survive the mission. Sensitivity $\left (\tfrac{TP}{TP + FN}\right )$ measures the model’s ability to assign enough maintenance to ensure subsystems that are going to fail during the mission actually survive. Specificity $\left (\tfrac{TN}{TN+FP}\right )$ assesses the model’s ability to properly identify subsystems that do not require any maintenance since they will survive the mission regardless. Negative predictive value $\left (\tfrac{TN}{TN+FN}\right )$ quantifies the model’s tendency to only avoid maintenance where it is non-critical. Accuracy $\left (\tfrac{TP + TN}{TP + TN + FP + FN}\right )$ is an overall metric that represents the model’s ability to assign enough maintenance only to subsystems that will fail and assign no maintenance to subsystems that will survive the mission (*i.e.,* properly assess the overall system’s critical and non-critical repair options and respond appropriately).

**Table 9:** Max-reliability optimal maintenance plan confusion matrix (with MCD sample size of $N=1000$)

	Adequate Maintenance	Inadequate/No Maintenance	
Subsystem Fails	*True Positives*	*False Negatives*	*Sensitivity*
	1.98 (66.0%)	0.00 (0.0%)	100.0%
Subsystem Survives	*False Positives*	*True Negatives*	*Specificity*
	0.54 (18.0%)	0.48 (16.0%)	47.1%
	*Precision*	*Negative Pred. Value*	*Accuracy*
	78.6%	100.0%	82.0%

**Table 10:** Min-cost optimal maintenance plan confusion matrix (with MCD sample size of $N=1000$)

	Adequate Maintenance	Inadequate/No Maintenance	
Subsystem Fails	*True Positives*	*False Negatives*	*Sensitivity*
	1.84 (61.3%)	0.14 (4.7%)	92.9%
Subsystem Survives	*False Positives*	*True Negatives*	*Specificity*
	0.22 (7.3%)	0.80 (26.7%)	78.4%
	*Precision*	*Negative Pred. Value*	*Accuracy*
	89.3%	85.1%	88.0%

As reported in Table 8, the reliability maximization model produces plans resulting in system survival for 50 of 50 trials (100%). This result shows a strong capability of ensuring system survival when that is the priority while also increasing maintenance time and cost when compared to the cost minimization model. The reliability maximization model ensures that subsystems in need of maintenance receive adequate repairs to survive the upcoming mission in nearly all cases, thus yielding high sensitivity and negative predictive values of 100%. However, the model also maintains subsystems that do not urgently require repairs for the upcoming mission, as demonstrated by the false positive value of 0.54 (18.0%) and specificity of 47.1%. This over-maintenance occurs because the model does not allow for any RUL prediction sample instance to result in mission failure, as shown by the average empirical reliability being equal to 1, leading to non-urgent maintenance. This demonstrates the model’s tendency to plan based on conservative estimates in cases where there is uncertainty about component/subsystem survival. However, despite this risk-aversion tendency, the model does not exhaust resources or replace all components, indicating it still recognizes some instances where maintenance is clearly non-urgent and does not result in empirical reliability improvements.

With the cost minimization model, 43 out of 50 trials (86%) result in survival. Furthermore, the average empirical system reliability is 95.74%. This result indicates that the cost minimization model also tends to make decisions based on conservative estimates when considering the reliability constraints. Such behavior is valuable when minimizing maintenance costs for a system where failures can be costly. Cost minimization classification metrics show a high sensitivity of 92.9%, resulting in very few instances of subsystems being inadequately maintained, though the model does incorrectly neglect some urgent subsystem maintenance in six trials, which is to be expected when minimizing costs based on uncertain survival. The cost minimization model also performs non-urgent maintenance on subsystems that are going to survive far less frequently than its reliability maximization counterpart. On average 0.22 (7.3%) subsystems are needlessly maintained in the cost minimization model, compared to 0.54 (18%) subsystems in the reliability maximization model. The fact that most trials result in survival with far fewer false positive instances indicates that the cost minimization model is very capable of identifying subsystems not urgently in need of repairs, thus demonstrating a strong ability to discern which maintenance actions yield the most benefit to the model.

In summary, these results highlight that the models are both capable of optimizing maintenance planning based on the empirical reliability distribution constraints and are often able to discern between components in urgent need of maintenance and those better left alone. Additionally, the high reliability in both cases indicates that this method tends to result in models that are more sensitive to component survival uncertainty, tending to use conservative estimates and performing more maintenance than is necessary to meet their desired level of reliability. Finally, it is important to underscore the swift runtime of this method. The problem posed is a moderately large system with k-out-of-n:G subsystems. Such a scenario can pose issues with computations due to the complexity of the reliability equations used in past works. Additionally, works like [6] have noted that DL models can suffer from long computation times. This method achieves strong performance while also delivering its results within less than two minutes, thus bypassing the computational challenges present in past literature [2, 32].

### Experiment 3: varying upcoming mission length

For the final experiment with the NASA C-MAPSS dataset, one of the 50 randomized trials generated for the second experiment is used as a test scenario where the upcoming mission length is varied between 30 and 120 cycles by increments of 10 to understand the effects on solution quality and model performance. Break duration is changed to $T_{0}=30$ and the budget to $C_{0}=80$. Hierarchical objective functions are also introduced to the SMP models to ensure that the reliability maximization model selects the plan with the lowest cost, and vice versa for the cost minimization model.

**Figure 9:**
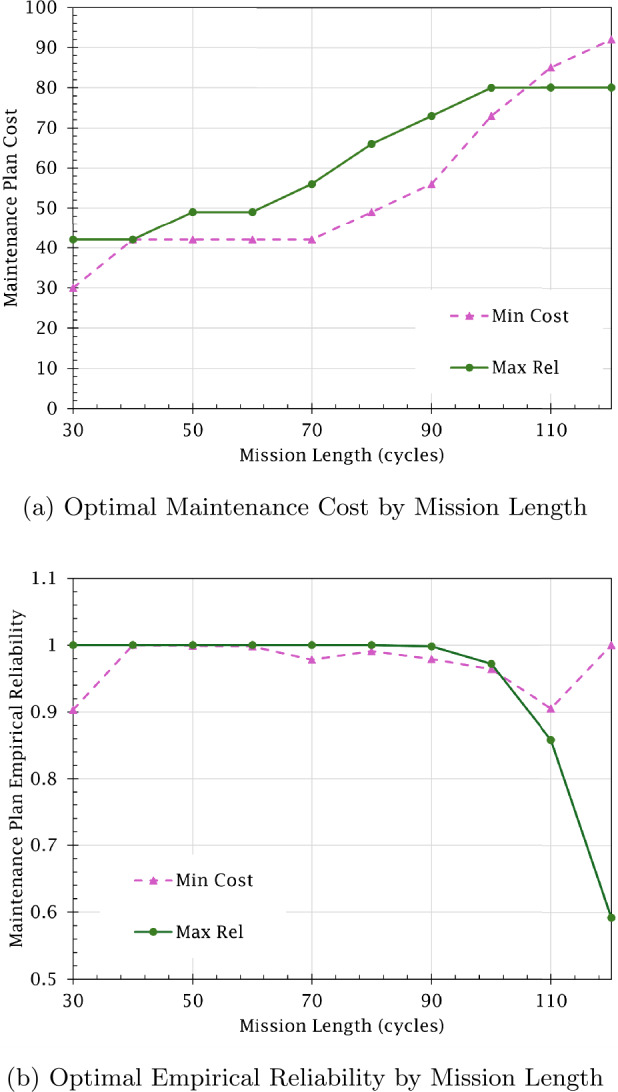
Maintenance plan quality when varying mission length (*U*)

Figs. 9a and 9b depict the changes in cost and reliability for the optimal maintenance plans selected for reliability maximization and cost minimization. The models behave very similarly, with both increasing resource use as mission length increases. The reliability maximization model uses more resources than the cost minimization model to ensure an empirical reliability of 1. Notably, once mission length exceeds 100 cycles, the cost minimization model begins requiring more resources to meet the required reliability threshold than are given to the reliability maximization model. In contrast, the reliability maximization model, having used all budget available (*i.e.,* maintenance cost has plateaued in Fig. 9a), produces plans resulting in empirical reliability decreasing sharply as mission length increases. These expected behaviors show that the integrated hybrid framework is yielding valid results.

**Figure 10:**
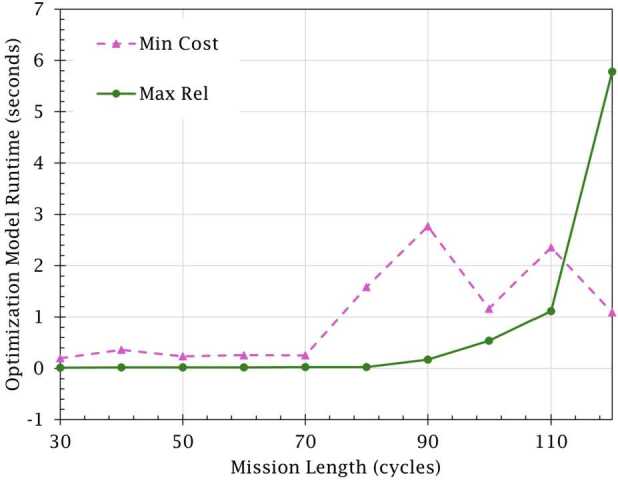
Optimization model runtime by mission length (*U*)

Finally, Fig. 10 depicts the computation times when solving both models. While the cost minimization model runtime is relatively stable, the reliability maximization model runtime increases exponentially as mission length increases. The reason for this behavior can likely be attributed to the model requiring more time to select its maintenance actions when more repairs are required to achieve maximum reliability, resulting in more maintenance action combinations to be evaluated. To further understand these models’ behavior, future research should also examine their sensitivity to changes in the break duration, budget, and minimum reliability threshold.

### Experiment 4: alternate dataset testing

To ensure that the method applies to more scenarios than simply the NASA C-MAPSS dataset, an alternative dataset is used in the same maintenance scenario as outlined in Sect. 5.3. The upcoming mission length is set to $U = 50$. The dataset selected concerns the approximation of the RUL of dust filters that are being exposed to various types of dust [11]. This dataset has been used in prior ML prognosis applications, such as in [59] and [60]. The testing dataset exposed 50 operating filters to dust until they failed and then pruned their data prior to failure while recording the RUL. Sensor data from the filters has been provided, along with operational setting details, such as the type of dust used. Given that sensor data would likely be the only information provided in an RUL prediction scenario, all operational data has been removed from the dataset for this experiment.

20 filters from the dataset are randomly selected to be used as the actual system component data, with the other 30 being used for model training and validation. The finalized DL model hyperparameters after tuning for this new dataset are as follows:

- Window length (*WL*) = 70 time steps
- Maximum RUL value = 75 cycles
- Number of epochs = 100
- Batch size = 128
- Training/Validation split = 75/25
- Number of CNN layers = 1
- Number of CNN layer filters = 128
- CNN layer kernel size = 7
- Number of Bi-LSTM layers = 2
- Number of hidden units in Bi-LSTM layers = [50, 10]
- Number of fully connected dense layers = 1
- Number of dense layer units = 200
- Dropout rate = 0.75
- Initial learning rate = 0.00005
- L2 regularization strength (*μ*) = 0.001
- Minimum MSE improvement required for adaptive learning rate = 10
- Adaptive learning rate patience = 10 epochs
- Adaptive learning rate reduction coefficient = 0.5
- Minimum permissible learning rate = $1\times 10^{-10}$

**Figure 11:**
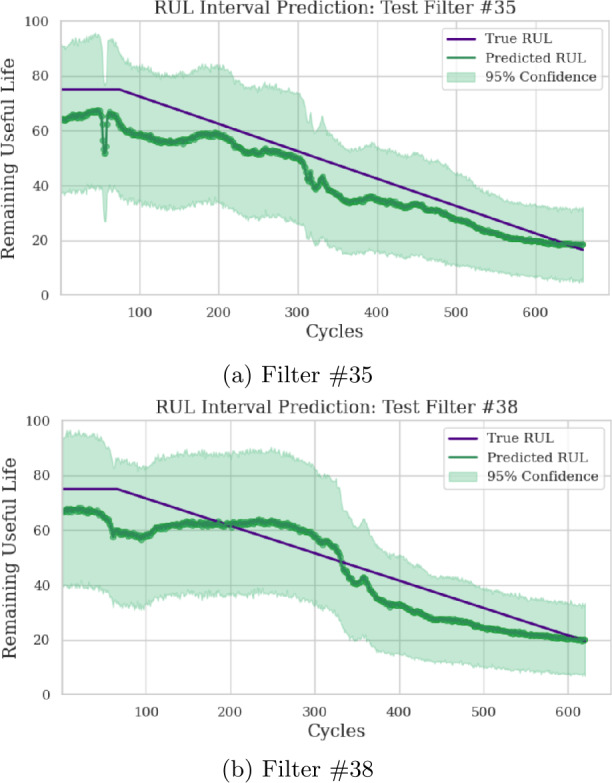
RUL point prediction accuracy visualization for two test filters

**Figure 12:**
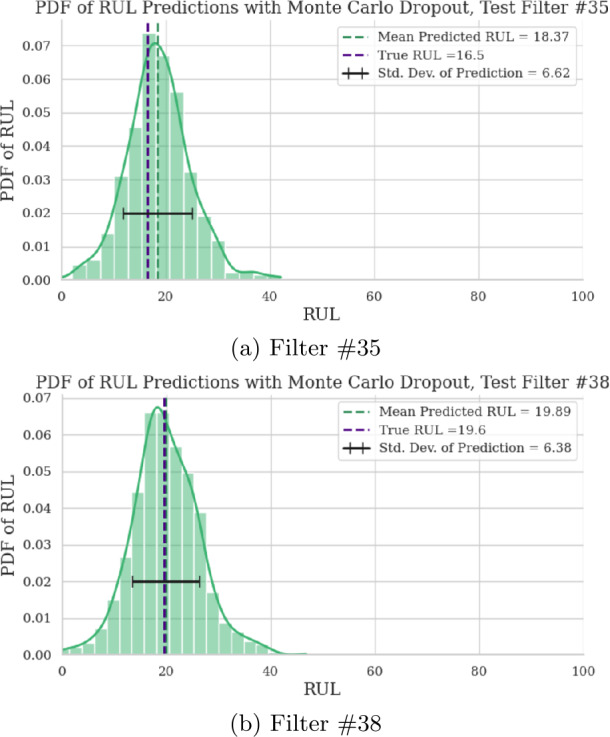
RUL prediction distribution for two test filters

After training the model, 50 trials are performed for SMP maintenance plan generation with the 20 filters not yet used. Each filter’s RUL is further pruned at a randomized point and its status is also randomized. Most of the same metrics used to evaluate the deep learning model for the C-MAPSS dataset are used for this experiment and are presented in Table 11. RUL point predictions and prediction distributions are provided for Filters 35 and 38 in Figs. 11 and 12, respectively. RMSE_C_, SC, and AC are not used, since those are specific to the C-MAPSS dataset.

**Table 11:** Deep learning model performance metrics with filter dataset

MAE	MSE	RMSE	Model Training Time (s)
7.57	97.87	9.89	448.7

Table 12 provides a comparison of prognostic ML model performance for various state-of-the-art works in prior papers. The model is able to achieve an MAE and RMSE better than other state-of-the-art models.

**Table 12:** State-of-the-art prognostic DL model comparison for filter dataset (best values bolded)

Model	MAE	RMSE
GPR [59]	20.50	26.40
Phys. based data generation [61]	–	10.40
ST-GS4D [60]	–	11.51
CNN-Bi-LSTM-Dense-MCD (this paper)	**7.57**	**9.89**

The average maintenance plan performance based on 50 randomized trials is presented in Table 13. Additionally, the confusion matrices for the reliability maximization and cost minimization maintenance plans are provided in Tables 14 and 15, respectively.

**Table 13:** Optimal maintenance plan metrics: Filter dataset (50 trials)

Objective Function	Average Cost	Average Duration	Average Empirical Reliability	Proportion of Trials Survived
Cost Min.	62.48	22.28	0.92	0.98
Rel. Max.	93.00	30.94	1.00	1.00

**Table 14:** Max-reliability optimal maintenance plan confusion matrix for filter dataset

	Adequate Maintenance	Inadequate/No Maintenance	
Subsystem Fails	*True Positives*	*False Negatives*	*Sensitivity*
	1.72 (57.3%)	0.00 (0.0%)	100.0%
Subsystem Survives	*False Positives*	*True Negatives*	*Specificity*
	1.28 (42.7%)	0.00 (0.0%)	0.0%
	*Precision*	*Negative Pred. Value*	*Accuracy*
	57.3%	–	57.3%

**Table 15:** Min-cost optimal maintenance plan confusion matrix for filter dataset

	Adequate Maintenance	Inadequate/No Maintenance	
Subsystem Fails	*True Positives*	*False Negatives*	*Sensitivity*
	1.70 (56.7%)	0.02 (0.7%)	98.8%
Subsystem Survives	*False Positives*	*True Negatives*	*Specificity*
	1.08 (36.0%)	0.20 (6.7%)	15.6%
	*Precision*	*Negative Pred. Value*	*Accuracy*
	61.2%	90.9%	63.3%

With this dataset, maintenance plans result in almost no instances of system failure during the mission, with only one instance encountered for the cost minimization plan, despite its use of significantly fewer resources than the reliability maximization model. When examining the confusion matrices, accuracy is at 57.3% for the reliability maximization model and 63.3% for the cost minimization model while the sensitivity remains extremely high. No false negatives are produced from reliability maximization and only 0.02 false negatives are produced from cost minimization. This phenomenon, paired with the high false positive rates, indicates that the uncertainty in RUL predictions tends to lead to the models taking a conservative approach and performing more maintenance than is necessary. This behavior is valuable in settings where failure is considered catastrophic. Additionally, this tendency to over-repair still results in the cost minimization model yielding plans with significantly lower resource usage when compared to the reliability maximization plans. This observation showcases that a tendency to over-repair does not inhibit the cost minimization model’s ability to lower resource use. These results confirm the proposed method’s applicability to various industrial settings and highlight its strengths.

## Contributions

To the best of our knowledge, this is the first paper to implement the concept of empirical distributions to compute system reliability when component RULs are predicted by DL models. Thus, the proposed framework is a valuable contribution towards the development of integrated ML/DL and maintenance planning methods that utilize the increasing availability of sensor data and ML technologies. This also represents a key step forward in developing accurate, efficient methods for modeling reliability in real-world systems via ML and DL methods. Such work sidesteps the computational challenges posed by classical, parametric methods while also avoiding potentially inaccurate assumptions about system reliability. This method also provides an integrated approach to maintenance planning, directly tying together ML prognostics and maintenance plan optimization, which is uncommon in the literature.

The numerical experiments performed in Sect. 5 demonstrate the strength of the proposed method. When attempting to solve the SMP with the C-MAPSS dataset for reliability maximization, the model is able to develop plans that result in system survival in almost all cases based on the RUL point predictions generated. This result highlights the accuracy of using ML/DL methods as the basis for empirical reliability distribution constraints.

The cost minimization formulation further emphasizes the accuracy of the DL predictions. While aiming for a minimum empirical reliability of 90%, the model’s average empirical reliability after optimization is over 95% and the model’s optimization plans result in mission survival in 86% of the trials. This demonstrates that the empirical reliability measure used in optimization is able to lead to system survival at an approximately equal rate, demonstrating its strong ability to represent a system’s actual reliability distribution. Furthermore, this tendency to prefer conservative estimates and aim for a higher reliability is beneficial when considering systems where failure is highly undesirable. Additionally, the cost minimization model achieves such results while rarely opting to maintain subsystems that are going to survive the upcoming mission, demonstrating how the DL predictions are accurate enough to inform the model on which components are most urgently in need of repairs.

It must also be highlighted that this paper builds significantly on the work of [3]. The popular CNN architecture is introduced to the DL model proposed by [3] to examine whether it can enhance model accuracy. Hyperparameter tuning is also undertaken to further refine method performance and ensure that optimal performance is achieved with CNN architecture to understand its impacts and whether it is worth implementing. This kind of tuning and comparison is invaluable when justifying the design of an ML model. The proposed SMP is also formulated to eliminate the need to calculate a parametric reliability, instead directly using RUL predictions to model an empirical reliability function. The proposed method is also tested on a dataset not considered by [3], thus demonstrating its applicability to various industrial contexts and ability to handle datasets other than C-MAPSS. The filter dataset has also never been used to generate maintenance plans, thus demonstrating additional utility in the dataset.

Finally, it must be emphasized that this data-driven method is able to completely circumvent traditional reliability modeling techniques. This approach omits the need to make potentially incorrect assumptions about component lifetimes and avoids the complex reliability equations that can result in computational challenges. The method also does not rely upon reinforcement learning or (meta)heuristic techniques to generate its maintenance plans, ensuring that the problem is solved to optimality based on the analyzed data. The method is validated with an experiment from [2], which considered assumed parametric distributions. The model is able to achieve near-optimal results within one minute of runtime when sampling from RUL distributions to build empirical approximations, rather than solving via exact reliability distribution modeling. The models developed in this paper are capable of yielding such promising results within approximately 70 seconds when using a DL model to analyze sensor data. This lack of reliability modeling assumptions, paired with the short runtime, highlights the model’s applicability to real-world scenarios with the only requirement being sensor data to train and test the model on.

## Conclusions

This paper develops a data-driven method for solving the SMP to optimality with empirical reliability constraints generated through a DL model using component sensor data. A hybrid model using CNN, Bi-LSTM, and dense neural network architecture is proposed to analyze time-series sensor data from system components and predict component RULs. MCD is then employed to generate a set of RUL predictions for each component. These predictions are converted into binary parameters used in empirical distribution constraints for modeling system reliability based on the upcoming mission length. The SMP is optimized under these constraints to either maximize the system’s empirical reliability or minimize total maintenance costs. With the additional augmentation of CNN architecture and the use of empirical reliability functions in the SMPs, this paper’s work builds significantly on that of [3]. Furthermore, the philosophy of hyperparameter tuning discussed in [9] is applied here to both refine the proposed DL model and demonstrate the impact of CNN architecture on the model designed by [3].

To test the performance of the proposed integrated method, four sets of numerical experiments are carried out. The first set of experiments concerns a scenario tested in [2] when employing assumed, parametric reliability distributions. The results of the first set of experiments validate the use of empirical reliability distribution functions in lieu of exact reliability functions. The second and third sets of experiments employ the NASA C-MAPSS dataset [10] in a series k-out-of-n:G structure. Numerous trial scenarios with randomized component statuses and ages are tested, and the upcoming mission length is also varied to understand SMP model behavior. The valid maintenance plans that result from these experiments show the pertinence and efficiency of the proposed framework. The final experiment uses an alternate dataset to show the method’s adaptability to other industrial contexts. Indeed, the maintenance plans generated are valid, often resulting in system survival based on conservative failure predictions. These results indicate that the proposed method can produce high-quality maintenance plans, even when faced with uncertain RUL predictions.

This work presents numerous opportunities for further development and expansion. Minimal hyperparameter tuning was done due to time constraints and strong early results. The accuracy values of 71.3% and 90% obtained were sufficient for the obtained valid maintenance plans, yet the hyperparameters can be better tuned (through *e.g.*, grid search method) to achieve more accurate RUL predictions. Moreover the optimization model can be modified to include uncertainty in maintenance and mission durations. Furthermore, as the primary goal of this paper is to showcase the capability of the proposed integrated model, the SMP considered does not include multiple missions. An interesting extension would be to consider the multi-mission SMP for large-scale systems and investigate the performance of the new framework when predicted RULs are longer than one or multiple mission lengths. A rolling horizon scheme could be implemented and tested. To further assess the method’s applicability and flexibility, datasets and scenarios from other industrial settings should be tested with it. Use of reinforcement learning and (meta)heuristics may be a valuable addition when applying the method to large-scale scenarios where computation times may become unreasonably high. Lastly, metrics such as confidence intervals and other uncertainty measures could be extracted from the DL model’s RUL estimates. There may be benefits in directly applying these uncertainty measures instead of using the individual point estimates.

## Data Availability

The NASA C-MAPSS Jet Engine Simulated Data [10] is an open-access dataset available on the NASA Open Data Portal at the following link: https://data.nasa.gov/Aerospace/CMAPSS-Jet-Engine-Simulated-Data/ff5v-kuh6/about_data. This article’s code is available upon request by contacting the corresponding author.
